# The effect of different transcranial magnetic stimulation protocols on pain scores in patients with neuropathic pain: a systematic review and meta-analysis

**DOI:** 10.3389/fpsyt.2026.1822963

**Published:** 2026-05-04

**Authors:** Zhicheng Zhu, Lisha Xie, Youjia Mao, Yong Fan, Cui Huang, Zijian Zhu, Nangen Song

**Affiliations:** Xinyu University, School of Physical Education, Xinyu, China

**Keywords:** meta-analysis, neuropathic pain, randomized controlled trial, repetitive transcranial magnetic stimulation, review

## Abstract

**Objective:**

To evaluate the effects of various transcranial magnetic stimulation (TMS) parameters—including stimulation duration, intensity, pulse number, frequency, and target region—on pain scores in patients with neuropathic pain (NP).

**Methods:**

Randomized controlled trials (RCTs) assessing the effects of TMS on NP were identified through searches of PubMed, Embase, Web of Science, and the Cochrane Library. Data were analyzed using Stata 18.0 with a random-effects model. Meta-regression, subgroup, sensitivity, and publication bias analyses were also performed.

**Results:**

A total of 14 studies met the eligibility criteria and were included in the meta-analysis. Overall, the pooled findings showed that rTMS was associated with significant improvements in pain outcomes, as reflected by reductions in VAS scores (SMD = -0.86, 95% CI: -1.51 to -0.22, P = 0.01) and SF-MPQ scores (MD = -5.79, 95% CI: -6.36 to -5.22, P < 0.001) in patients with NP. By contrast, no significant effect was detected for NRS scores (MD = -0.41, 95% CI: -1.07 to 0.25, P = 0.22). Further subgroup analyses suggested that studies characterized by a shorter intervention period (<2 weeks), fewer total pulses (<2000), higher stimulation frequency (≥10 Hz), and lower motor threshold (<90% RMT) were more likely to report significant reductions in VAS scores. Nevertheless, none of these subgroup comparisons demonstrated a statistically significant difference when compared with their respective reference categories, including intervention period ≥2 weeks, total pulse number ≥2000, stimulation frequency <10 Hz, and motor threshold ≥90% RMT. In contrast, stimulation delivered to non-M1 targets also produced a significant decrease in VAS scores, and the between-subgroup comparison indicated a significant difference relative to stimulation over the M1 region.

**Conclusion:**

rTMS is effective in reducing VAS pain scores in patients with NP. However, given the limited sample size, further large-scale randomized controlled trials are needed to confirm these results.

**Systematic review registration:**

https://www.crd.york.ac.uk/prospero/, identifier CRD420251013132.

## 1 Introduction

Neuropathic pain (NP) is a prevalent chronic pain condition resulting from disorders affecting the central and peripheral somatosensory nervous systems (1, 2). Epidemiological data indicate that the prevalence of NP in the general population ranges from 7% - 10% (3), reaching 9.8% among community-dwelling adults (4). Furthermore, NP is more common among women, middle-aged and elderly individuals, illiterate populations, manual laborers, rural residents, and those with lower socioeconomic status (5, 6). Clinical studies have demonstrated that NP patients experience not only persistent pain but also sensory abnormalities, including spontaneous tingling, cold allodynia, refractory itching, and characteristic numbness (7). Notably, in some patients, applying pressure to numb areas can elicit severe electric shock–like or burning pain (7, 8). These complex symptoms profoundly impair physical function, restrict daily activities, increase the risk of disability, and substantially reduce quality of life (9).

Although pharmacological treatments—such as anticonvulsants and tricyclic antidepressants—remain the primary interventions for NP, their clinical efficacy is limited. Approximately 30%–50% of patients exhibit treatment resistance, and long-term medication use may lead to reduced tolerance and dose-dependent adverse effects (10–12). Therefore, identifying safe and effective therapeutic alternatives is essential for NP management (13). Repetitive transcranial magnetic stimulation (rTMS), a safe, non-invasive neuromodulation technique, stimulates cortical neurons via electromagnetic induction, modulating neuronal excitability and neuroplasticity (14). This mechanism contributes to its significant analgesic effects in NP patients. Numerous studies have demonstrated that rTMS effectively reduces pain scores and alleviates pain symptoms in this population (15–17).

Although rTMS has shown clinical potential in treating NP, controversy persists regarding the optimal stimulation parameters, particularly concerning stimulation frequency. Jin et al. (18) conducted a meta-analysis and found that 5 Hz, 10 Hz, and 20 Hz stimulations all alleviated pain in NP patients. Similarly, a meta-analysis by Attia et al. (19) suggested that the most effective parameters for inducing analgesia were a stimulation frequency of 10–20 Hz, intensity at 80%–120% of the resting motor threshold (RMT), 1,000–2,000 pulses, and 5–10 treatment sessions. In contrast, Jiang et al. (20) reported that rTMS at frequencies ≥5 Hz effectively relieved NP. However, previous systematic reviews have focused only on specific parameters—such as stimulation site, frequency, intensity, pulse number, and treatment sessions—without comprehensively analyzing the analgesic effects of different stimulation parameters or intervention period. These parameters are critical to rTMS protocol design, and omission of any factor may significantly affect its therapeutic efficacy in NP patients. Therefore, further research is urgently needed to provide more comprehensive evidence.

In summary, to address the above clinical controversies and determine the optimal rTMS stimulation parameters, this study aims to evaluate the effects of rTMS compared with sham stimulation on pain outcomes in patients with NP. Specifically, it investigates the influence of key parameters—including intervention period, motor threshold, pulse number, stimulation frequency, and target site—on pain outcomes in NP patients. The findings of this study are expected to provide evidence-based guidance for clinical practice and inform optimal rTMS treatment protocols for NP.

## 2 Method

This study was conducted in accordance with the Preferred Reporting Items for Systematic Reviews and Meta-Analyses (PRISMA) guidelines, and the study protocol was registered in PROSPERO (CRD420251013132).

### 2.1 Inclusion and exclusion criteria for systematic reviews

#### 2.1.1 Study types

Randomized controlled trials (RCTs) evaluating repetitive transcranial magnetic stimulation (rTMS), in patients with NP were included. Cohort studies, observational studies, and other non-randomized trials were excluded. No restrictions were applied regarding language, publication date, or publication status.

#### 2.1.2 Participant types

Patients of any age with NP or participants receiving rTMS were included. Animal studies were excluded from this review.

#### 2.1.3 Intervention types

Participants in the intervention group received rTMS, while those in the control group received sham stimulation. Studies involving non-TMS interventions—such as transcranial direct current stimulation, deep brain stimulation, and vagus nerve stimulation—or those in which the control group did not receive sham stimulation were excluded.

#### 2.1.4 Outcome types

The primary outcome measure was the Visual Analogue Scale (VAS). Secondary outcomes included the Numerical Rating Scale (NRS) and the Short-Form McGill Pain Questionnaire (SF-MPQ). Studies using the Brief Pain Inventory (BPI), Beck Depression Inventory (BDI), or Hospital Anxiety and Depression Scale (HADS) as primary outcomes were excluded. Additionally, studies lacking sufficient data to calculate effect sizes, and for which attempts to contact the corresponding authors were unsuccessful, were excluded.

### 2.2 Search methods for study identification

We conducted a comprehensive search of PubMed, Embase, Web of Science, and the Cochrane Library using a combination of MeSH terms and keywords, with the final search performed on February 18, 2025. The primary search terms included “transcranial magnetic stimulation” and “neuralgia,” aiming to identify RCTs evaluating the effects of TMS on patients with NP. Additionally, existing reviews, systematic reviews, and meta-analyses were screened to identify further relevant studies.

### 2.3 Data extraction and management

Two reviewers (LS X and YJ M) independently assessed the eligibility of the studies. Initially, studies were excluded based on their titles and abstracts, with reasons for exclusion documented. Full texts of the remaining studies were then retrieved and independently reviewed by both reviewers. Data were extracted using standardized forms, including first author, publication year, sample size, participants’ age and sex, intervention type, intervention period, intervention frequency, motor threshold, stimulation site, and total pulse number. The outcome measures assessed immediately after completion of treatment were consistently extracted in this study. For studies in which outcome data were presented in graphical form, two reviewers independently extracted the data from the figures using GetData 2.22 software, and the average of the two extracted values was used to reduce measurement error. An overall assessment of the included studies was subsequently performed. Disagreements were resolved by re-examining the full texts and discussing with a third reviewer (ZC Z) until consensus was reached.

### 2.4 Risk of bias assessment

The risk of bias was assessed in accordance with the Cochrane Handbook for Systematic Reviews of Interventions, with any disagreements resolved through consensus. We evaluated the risk of bias as high, low, or unclear across the following domains: selection bias (random sequence generation and allocation concealment), performance bias (blinding of participants and personnel), detection bias (blinding of outcome assessors), attrition bias (incomplete outcome data), reporting bias (selective outcome reporting), and other potential sources of bias. When necessary, study authors were contacted to clarify concerns related to bias. Risk of bias summary and domain-specific graphs were generated using Review Manager 5.4.

### 2.5 Data analysis

Statistical analyses were performed using Stata 18.0. Mean difference (MD) with 95% confidence intervals (95% CI) served as the effect size measure. For the same outcome measured using different units, the standardized mean difference (SMD) was used for pooled analysis. Heterogeneity among studies was assessed using the I² statistic and the Q test P-value. When I² was less than 50% and P ≥ 0.1, heterogeneity was considered low, and a fixed-effects model was applied to estimate the pooled effect size. Conversely, when I² exceeded 50% and P < 0.1, heterogeneity was deemed high, warranting the use of a random-effects model. For studies exhibiting significant heterogeneity, meta-regression analyses were conducted to explore potential sources. Subgroup analyses were performed to identify optimal rTMS stimulation parameters, stratified by intervention period (≥2 weeks vs. <2 weeks), total pulse number (≥2000 vs. <2000), stimulation frequency (>10 Hz vs. ≤10 Hz), motor threshold (≥90% RMT vs. <90% RMT), and stimulation site (M1 area vs. other regions). Sensitivity analyses employing a leave-one-out method evaluated the robustness of the results by assessing the influence of individual studies. Publication bias was assessed using Egger’s test, with a P-value > 0.05 indicating no significant bias.

## 3 Results

### 3.1 Literature search results

**Figure 1:**
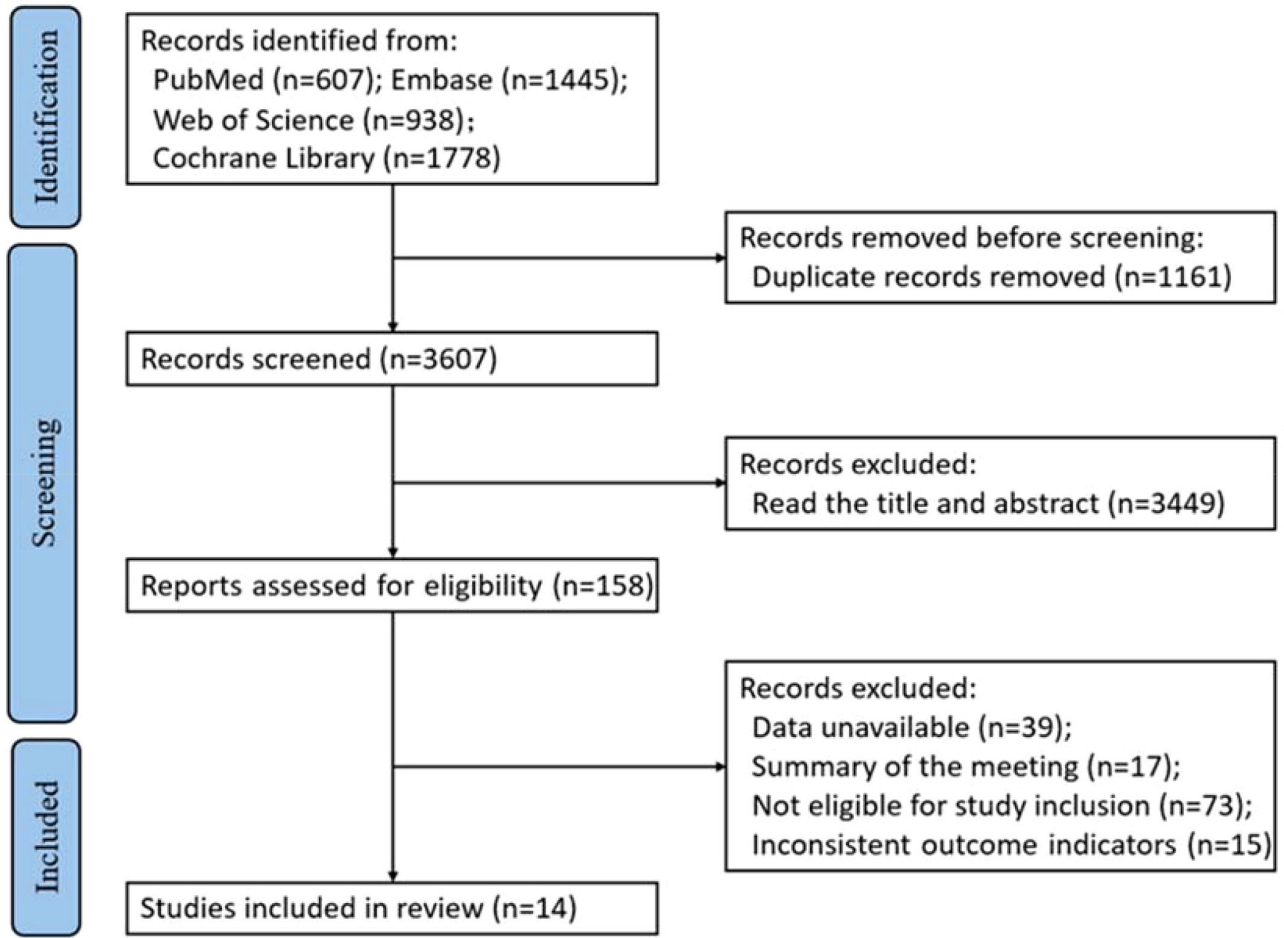
Literature screening flowchart.

A total of 4,768 records were identified through searches of PubMed, Embase, Web of Science, and the Cochrane Library. After removing 1,161 duplicates using EndNote, 3,607 records remained. Title and abstract screening excluded 3,449 records, resulting in 158 articles for full-text review. Among these, 144 articles were excluded for the following reasons: 39 due to insufficient data for extraction, 73 for failing to meet inclusion criteria, 15 due to incompatible outcome measures, and 17 being conference abstracts. Ultimately, 14 studies were included in the meta-analysis. The literature screening process is depicted in **Figure 1**.

### 3.2 Characteristics of the literature

Among the 14 included studies, the intervention method was described for the experimental group in all studies, all of which used rTMS (14, 17, 21–32), while the control group received sham stimulation in all cases. Among these studies, 8 targeted the M1 region as the stimulation site (17, 21, 23, 24, 26, 27, 31, 32). Additionally, 8 studies applied a stimulation frequency of 10 Hz (17, 21, 24–27, 29, 30) (**Table 1**).

**Table 1:** Basic information on the included literature.

Author	Year	N (E/C)	Type of intervention		Intervention period	Pulse number	rTMS site	Frequency	Motor threshold	Sham procedure type	Outcome
			E	C							
Ambriz-Tututi, M (23).	2016	67 (41/26)	rTMS	Sham rTMS	1 week	/	M1/S1	20 Hz	95% RMT	Tilted Coil	SF-MPQ
Zhao, C. G (17).	2020	48 (24/24)	rTMS	Sham rTMS	3 weeks	1500	M1	10 HZ	90% RMT	Sham coil	NRS, SF-MPQ
Zhao, C.-G (24).	2021	38 (19/19)	rTMS	Sham rTMS	3 weeks	1500	M1	10 HZ	80% RMT/ 100% RMT	Sham coil	NRS, SF-MPQ
Yílmaz, B (25).	2014	16 (9/7)	rTMS	Sham rTMS	10 days	1500	/	10 HZ	110% RMT	Tilted Coil	VAS
Kang, B. S (26).	2009	24 (13/11)	rTMS	Sham rTMS	5 days	/	M1	10 HZ	80% RMT	Tilted Coil	NRS
Bursali, C (14).	2021	20 (10/10)	rTMS	Sham rTMS	10 days	1000	/	5 Hz	70% RMT	Sham coil	VAS
Farnes, N (27).	2024	29 (14/15)	rTMS	Sham rTMS	3 weeks	3000	M1	10 HZ	80% RMT	Sham coil	NRS
Ahmed, M. A (28).	2011	27 (17/10)	rTMS	Sham rTMS	/	200	motor cortical	20 HZ	80% RMT	Tilted Coil	VAS
Lefaucheur, J. P (29).	2001	16 (6/10)	rTMS	Sham rTMS	/	/	motor cortical	10 HZ	80% RMT	Sham coil	VAS
Borckardt, J. J (30).	2006	20 (10/10)	rTMS	Sham rTMS	/	4000	/	10 HZ	100% RMT	Sham coil	VAS
Hosomi, K (31).	2020	142 (72/70)	rTMS	Sham rTMS	4 weeks	/	M1	5 HZ	90% RMT	Sham coil	VAS, SF-MPQ
Khedr, E. M (32).	2014	34 (17/17)	rTMS	Sham rTMS	10 days	2000	M1	20 HZ	80% RMT	Tilted Coil	VAS
H. Wang (21).	2023	30 (20/10)	rTMS	Sham rTMS	2 weeks	3000	M1	10 HZ	100% RMT	Sham coil	VAS
H. Wang (21).	2023	30 (20/10)	rTMS	Sham rTMS	2 weeks	3000	DLPFC	10 HZ	100% RMT	Sham coil	VAS
D. Vats (22).	2024	19 (10/9)	rTMS	Sham rTMS	2 weeks	1200	DLPFC	1 HZ	90% RMT	Tilted Coil	VAS

### 3.3 Evaluation of risk bias

**Figure 2:**
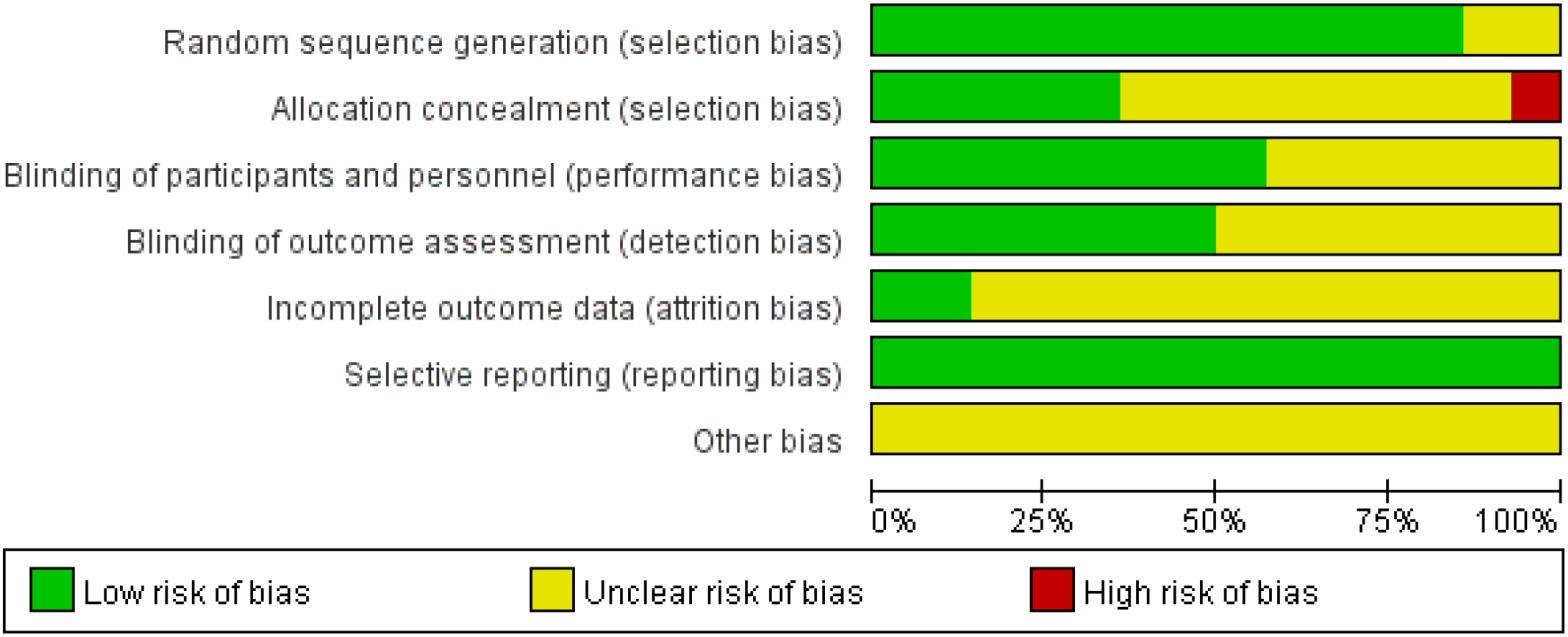
Risk of bias graph: review authors' judgements about each risk of bias item presented as percentages across all included studies.

**Figure 3:**
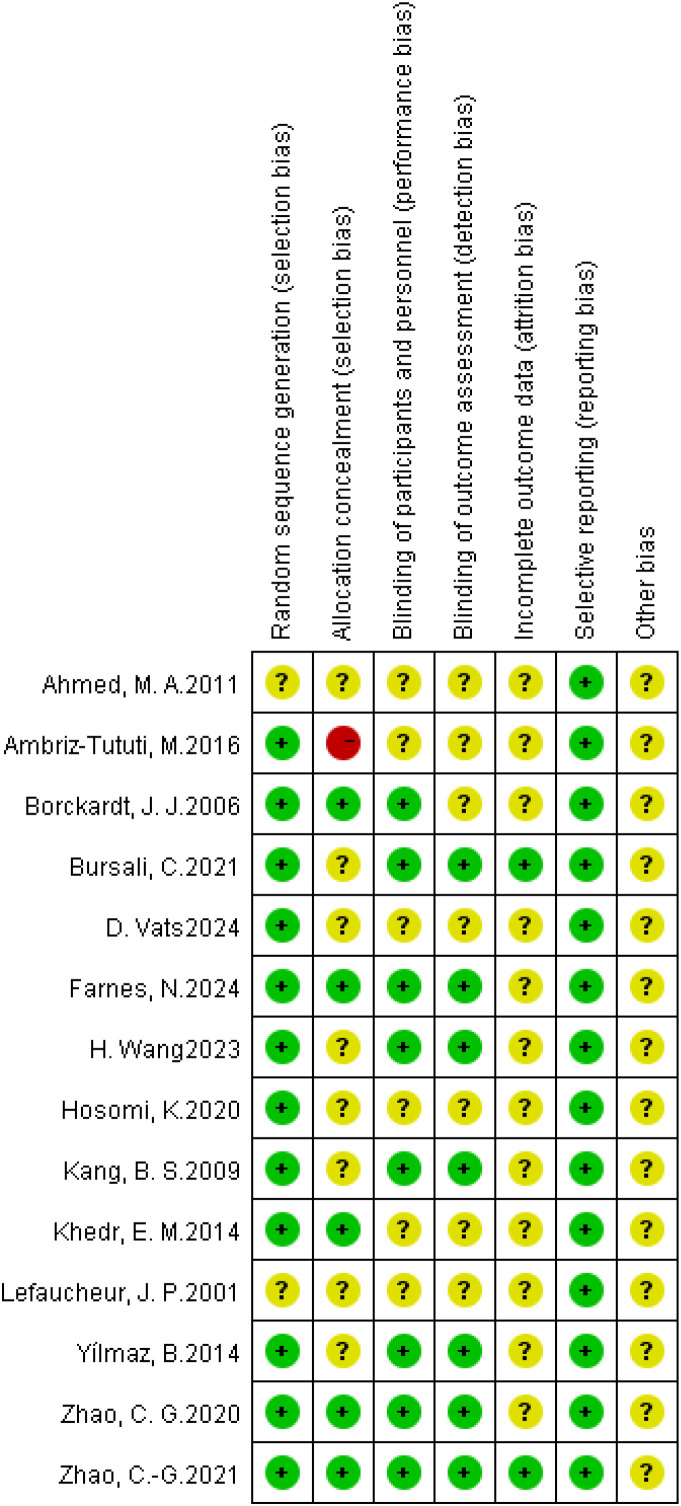
Risk of bias summary: review authors’ judgements about each risk of bias item for each included study.

Among the 15 studies, 13 explicitly described their randomization methods (14, 17, 21–27, 30–33), while 2 did not clearly report their randomization procedures (28, 29). Only 5 studies explicitly mentioned whether allocation concealment was implemented (17, 24, 27, 30, 32). 8 studies employed a double-blind design (14, 17, 21, 24–27, 33), whereas the remaining studies did not use double blinding. All studies reported the predefined outcome measures (**Figures 2**, **3**).

### 3.4 The effect of rTMS on VAS scores in patients with NP

**Figure 4:**
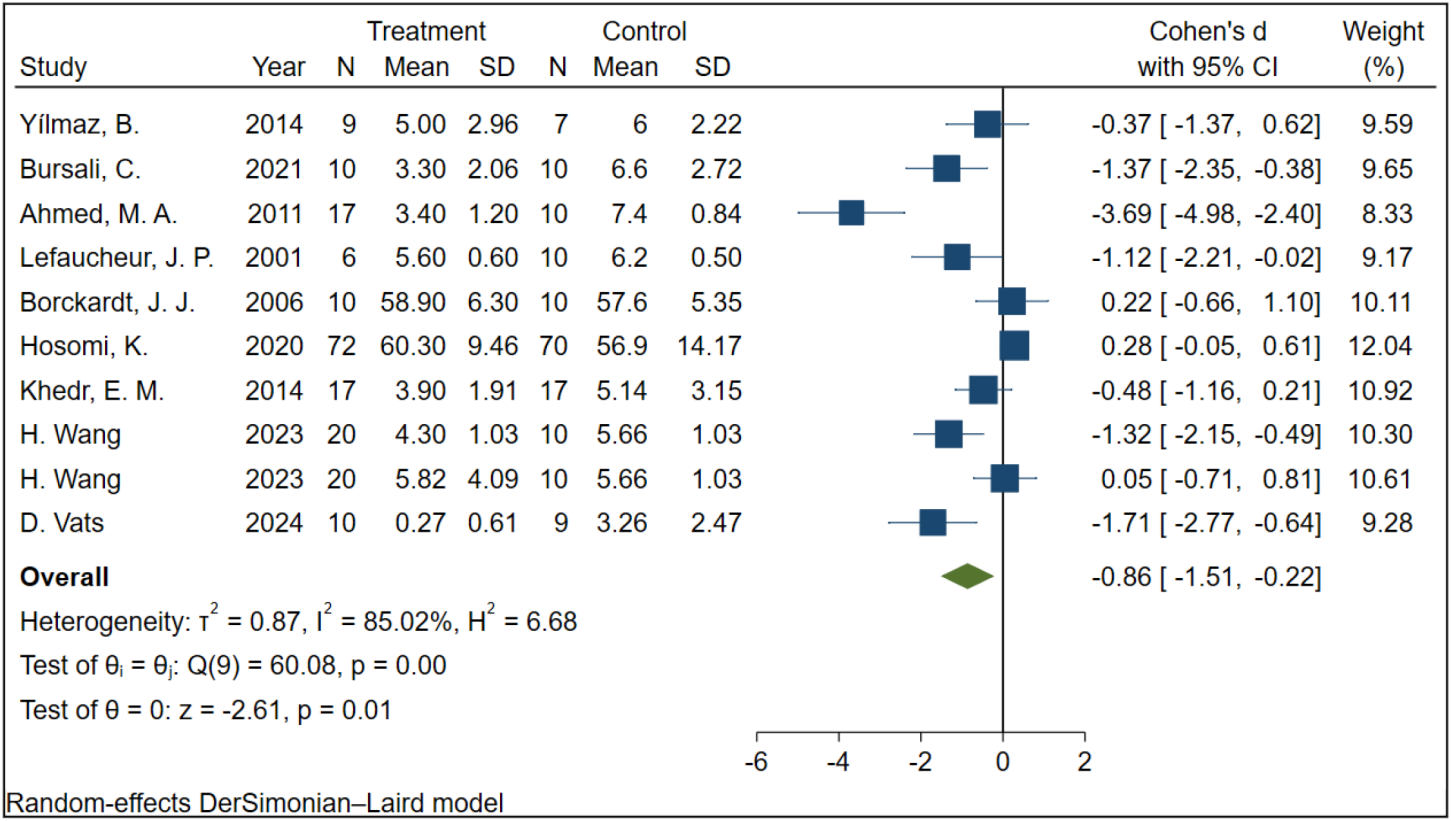
The effect of rTMS on VAS in NP patients.

A total of 9 studies involving 334 participants examined the effect of rTMS on VAS scores in patients with NP. Due to significant heterogeneity among studies (I² = 85.02%, P < 0.001), a random-effects model was employed. The results indicated that, compared to the sham stimulation group, rTMS significantly reduced VAS scores in NP patients (SMD = -0.86, 95% CI: -1.51 to -0.22, P = 0.01) (**Figure 4**).

### 3.5 Secondary outcome meta-analyses

#### 3.5.1 The effect of rTMS on NRS scores in patients with NP

**Figure 5:**
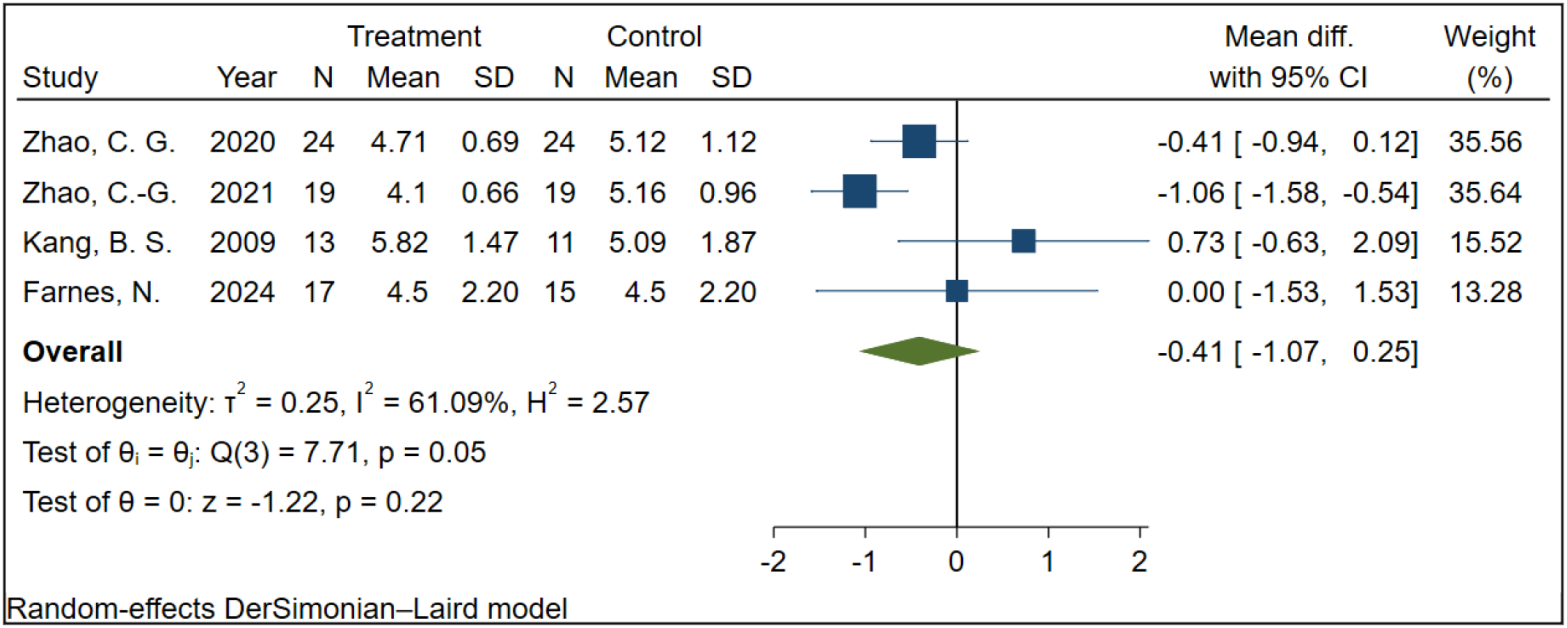
The effect of TMS on NRS in NP patients.

A total of 4 studies involving 142 participants examined the effect of rTMS on NRS scores in patients with NP. Due to significant heterogeneity among studies (I² = 61.09%, P = 0.05), a random-effects model was employed. The results indicated that, compared with the sham stimulation group, TMS did not have a statistically significant effect on NRS scores in NP patients (MD = -0.41, 95% CI: -1.07 to 0.25, P = 0.22) (**Figure 5**).

#### 3.5.2 The effect of rTMS on SF-MPQ scores in patients with NP

**Figure 6:**
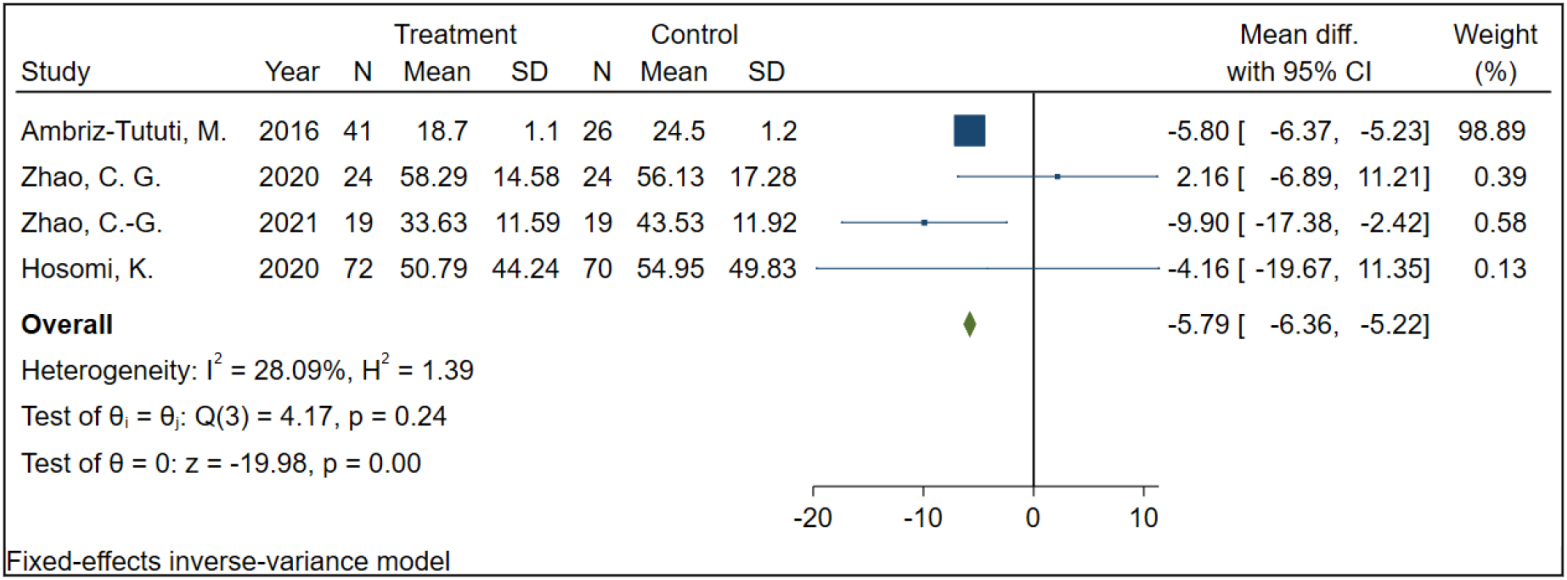
The effect of rTMS on SF-MPQ in NP patients.

A total of 4 studies involving 295 participants examined the effect of rTMS on SF-MPQ scores in patients with NP. Given the low heterogeneity among the included studies (I² = 28.09%, P = 0.24), a fixed-effect model was used for the analysis. The results indicated that, compared with the sham stimulation group, rTMS significantly reduced SF-MPQ in NP patients (MD = -5.79, 95% CI: -6.36 to -5.22, P < 0.001) (**Figure 6**).

### 3.6 Regression analysis

Meta-regression analyses were performed to identify potential sources of heterogeneity for the primary outcome measure. These analyses examined intervention frequency (>10 Hz vs. ≤10 Hz), motor threshold (≥90% RMT vs. <90% RMT) and sham stimulation type (sham coil vs tilted coil) as possible influencing factors. The results indicated that neither intervention frequency, intervention intensity, nor sham stimulation type significantly contributed to the heterogeneity of VAS scores (P > 0.05) (**Table 2**).

**Table 2:** The results of regression analysis of VAS.

Parameter	Coef	T	P
Frequency	-0.14	-0.18	0.861
Motion threshold	-0.99	-1.33	0.233
Sham Procedure Type	-0.78	-1.04	0.337

### 3.7 Subgroup analysis

To further investigate the effect of rTMS on VAS scores, subgroup analyses were performed according to intervention period, total pulse number, stimulation frequency, motor threshold, and stimulation site. As shown in **Table 3**, an intervention period of ≥2 weeks had no statistically significant effect on VAS scores (MD = -0.60, 95% CI: -1.54 to 0.35, P = 0.215), whereas an intervention period of <2 weeks was associated with a statistically significant reduction in VAS scores (MD = -0.69, 95% CI: -1.26 to -0.12, P = 0.017); however, the difference between the two subgroups was not statistically significant (P = 0.869). Total pulse number ≥2000 had no statistically significant effect on VAS scores (MD = -0.38, 95% CI: -1.02 to 0.26, P = 0.239), whereas total pulse number <2000 significantly reduced VAS scores (MD = -1.74, 95% CI: -2.98 to -0.49, P = 0.006), with no statistically significant difference between the two subgroups (P = 0.059). Stimulation frequency <10 Hz had no statistically significant effect on VAS scores (MD = -0.86, 95% CI: -2.29 to 0.57, P = 0.236), whereas stimulation frequency ≥10 Hz significantly reduced VAS scores (MD = -0.88, 95% CI: -1.67 to -0.09, P = 0.028), and the difference between the two subgroups was not statistically significant (P = 0.985). Motor threshold ≥90% RMT had no statistically significant effect on VAS scores (MD = -0.41, 95% CI: -1.06 to 0.24, P = 0.218), whereas motor threshold <90% RMT significantly reduced VAS scores (MD = -1.59, 95% CI: -2.82 to -0.36, P = 0.011), with no statistically significant difference between the two subgroups (P = 0.097). Stimulation at the M1 region had no statistically significant effect on VAS scores (MD = -0.31, 95% CI: -0.99 to 0.37, P = 0.372), whereas stimulation at other sites significantly reduced VAS scores (MD = -2.13, 95% CI: -3.57 to -0.70, P = 0.004), and the difference between the two subgroups was statistically significant (P = 0.024).

**Table 3:** Subgroup analysis of rTMS on VAS in patients with NP.

Parameter	Group	Participants (E/C)	MD (95% CI)	P (Overall Effect)	Heterogeneity		P (Group difference)
					P	I^2^, %	
Intervention Period	≥2 weeks	(122/99)	-0.60 (-1.54, 0.35)	0.215	<0.001	86.4	0.869
	<2 weeks	(36/34)	-0.69 (-1.26, -0.12)	0.017	0.275	22.5	
	Total	(158/133)	-0.63 (-1.24, -0.02)	0.044	<0.001	79.6	
Pulse Number	≥2000	(67/47)	-0.38 (-1.02, 0.26)	0.239	0.047	62.4	0.059
	<2000	(46/36)	-1.74 (-2.98, -0.49)	0.006	0.001	81.4	
	Total	(113/83)	-1.01 (-1.74, -0.28)	0.007	<0.001	80.8	
Frequency	≥10 HZ	(99/74)	-0.88 (-1.67, -0.09)	0.028	<0.001	81.3	0.985
	<10 HZ	(92/89)	-0.86 (-2.29, 0.57)	0.236	<0.001	90.0	
	Total	(191/163)	-0.86 (-1.51, -0.22)	0.009	<0.001	85.0	
Motor threshold	≥90%RMT	(141/116)	-0.41 (-1.06, 0.24)	0.218	<0.001	78.2	0.097
	<90%RMT	(50/47)	-1.59 (-2.82, -0.36)	0.011	<0.001	84.1	
	Total	(191/163)	-0.86 (-1.51, -0.22)	0.009	<0.001	85.0	
rTMS site	M1	(129/107)	-0.31 (-0.99, 0.37)	0.372	0.003	79.0	0.024
	Other	(33/29)	-2.13 (-3.57, -0.70)	0.004	0.009	78.8	
	Total	(162/136)	-1.04 (-1.89, -0.18)	0.017	<0.001	88.9	

### 3.8 Sensitivity analysis

**Figure 7:**
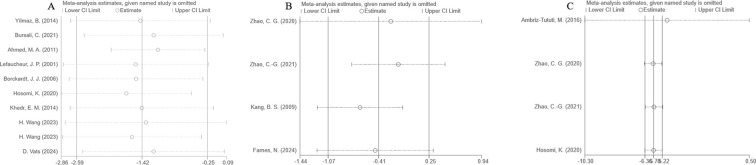
**(A)** VAS scores, **(B)** NRS scores, **(C)** SF-MPQ scores.

Sensitivity analyses were performed using STATA 18.0. For VAS and NRS, the MD values obtained after excluding each individual study did not exceed the 95% CI of the overall MD, indicating robust results. However, the SF-MPQ results appeared less robust, as excluding the study by Ambriz-Tututi, M. et al. (23) had a substantial impact on the overall effect estimate (**Figure 7**).

### 3.9 Publication bias detection

**Figure 8:**
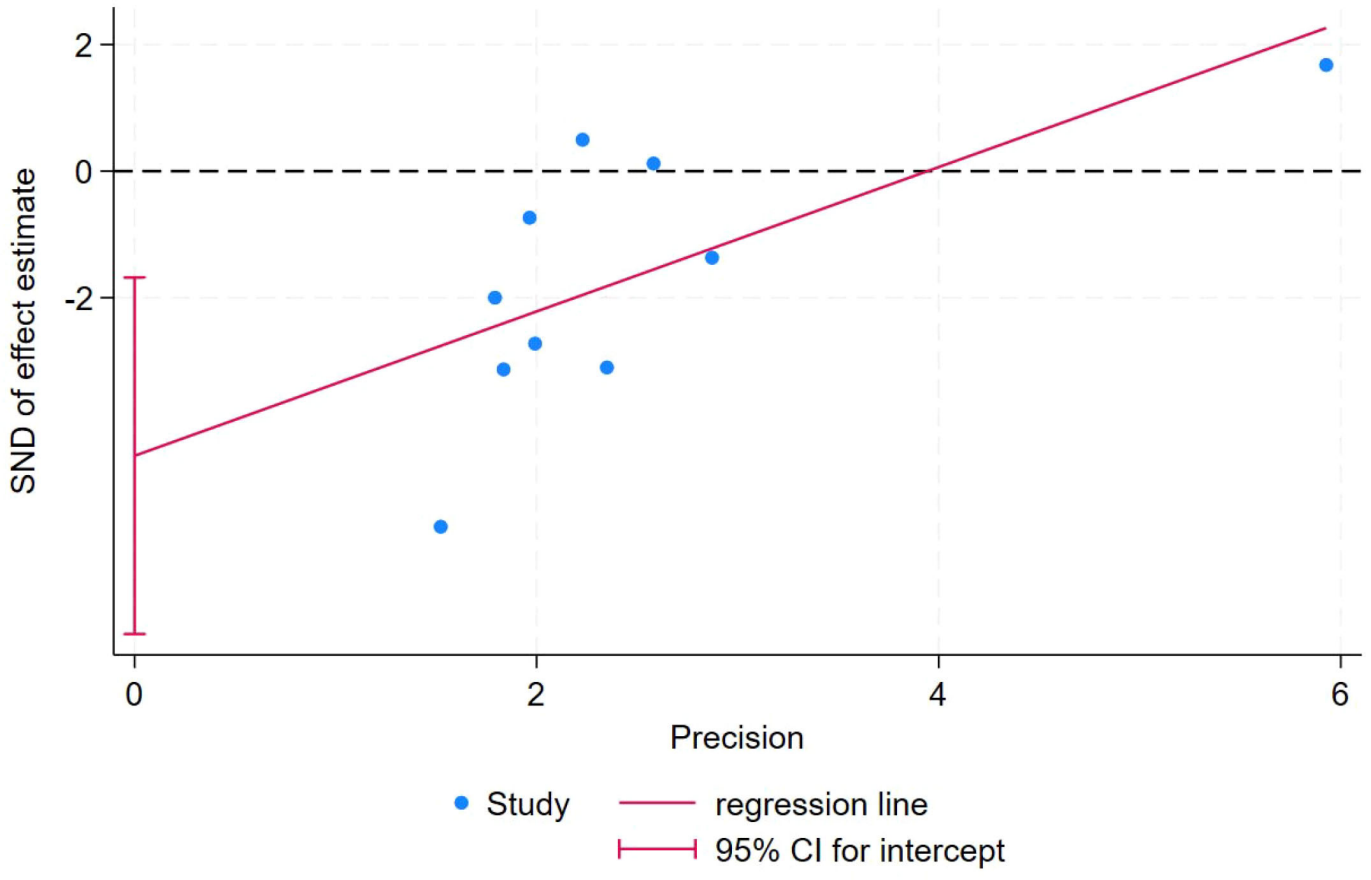
rTMS publication bias for VAS in NP patients.

Publication bias for the primary outcome was assessed using Egger’s test, and the results indicated significant evidence of publication bias for VAS (P = 0.006) (**Figure 8**).

## 4 Discussion

This study focused on the effects of rTMS on pain-related outcomes, including VAS, NRS, and SF-MPQ scores, in patients with NP. The pooled findings demonstrated that rTMS was associated with significant improvements in VAS and SF-MPQ scores, whereas no significant benefit was observed for NRS scores. To investigate possible sources of heterogeneity in the VAS outcome, meta-regression analyses were performed with stimulation frequency, motor threshold, and sham stimulation type entered as covariates. None of these variables was found to significantly account for the between-study heterogeneity. The residual heterogeneity may instead be related to differences in the underlying etiology of NP. The included studies involved both peripheral NP conditions, such as postherpetic neuralgia, malignancy-related NP, and phantom limb pain (21, 28, 32), and central neuropathic pain conditions, including pain following spinal cord injury and central post-stroke pain (17, 24). Because NP arising from different etiologies may vary in pathophysiological basis, clinical manifestations, and responsiveness to rTMS, these differences may have contributed substantially to the observed heterogeneity across studies. Variability in the number of treatment sessions may also have played a role. However, these factors were not incorporated into the meta-regression because relevant data could not be extracted from several included studies. To further assess whether treatment parameters influenced analgesic outcomes, subgroup analyses of VAS were conducted according to intervention period, total pulse number, stimulation frequency, motor threshold, and stimulation site. Significant reductions in VAS scores were observed in studies with an intervention period of <2 weeks, a total pulse number of <2000, a stimulation frequency of ≥10 Hz, and a motor threshold of <90% RMT. Nevertheless, none of these effects differed significantly from those observed in the corresponding comparator subgroups, namely intervention period ≥2 weeks, total pulse number ≥2000, stimulation frequency <10 Hz, and motor threshold ≥90% RMT. By contrast, stimulation delivered to non-M1 sites not only produced a significant reduction in VAS scores, but also showed a significantly different effect when compared with stimulation targeting the M1 region.

In the present study, rTMS was associated with significant improvements in VAS and SF-MPQ scores in patients with NP compared with sham stimulation, indicating a beneficial effect on pain relief. These findings are in line with previous reports by Gao et al. (34) and Jiang et al. (20). As a noninvasive neuromodulatory intervention, rTMS has demonstrated promising therapeutic value in the management of NP and may offer short-term analgesic benefits (35, 36). The potential analgesic effects of rTMS may be explained by several neurophysiological mechanisms. First, rTMS may modulate the neuroinflammatory milieu by increasing the expression of anti-inflammatory cytokines, such as IL-10 and IL-4, while suppressing proinflammatory cytokines, thereby improving synaptic plasticity and stabilizing neuronal excitability (2, 37, 38). Second, the time-varying magnetic field generated by rTMS induces electric currents within neural tissue, which can directly alter cortical excitability (39). Third, in patients with NP, such modulation of neuronal activity may help normalize aberrant signaling within pain-processing pathways, ultimately contributing to pain alleviation (40). In contrast, no statistically significant improvement was detected in NRS scores following rTMS treatment. This discrepancy may, at least in part, be related to differences in stimulation targets. Andre-Obadia et al. (41), for example, reported that therapeutic responses may vary according to the stimulation site in patients with NP. Nevertheless, there is still no standardized strategy for determining the optimal stimulation target, and current evidence remains insufficient to establish the most effective stimulation protocol (19). Taken together, these findings suggest that individualized stimulation protocols may be important in clinical practice to maximize the therapeutic benefits of rTMS for patients with NP.

This study further examined the influence of different rTMS parameters on VAS outcomes in patients with NP through subgroup analyses. The findings suggested that studies characterized by an intervention period of <2 weeks, a total pulse number of <2000, a stimulation frequency of ≥10 Hz, a motor threshold of <90% RMT, and stimulation at non-M1 sites were more likely to report significant reductions in VAS scores. Nevertheless, except for stimulation site, the tests for subgroup differences were not statistically significant, and these findings should therefore be interpreted cautiously. With respect to intervention period, a duration of <2 weeks was associated with a significant improvement in VAS scores, whereas no significant effect was observed in the ≥2-week subgroup. One possible explanation is that shorter treatment courses may reduce treatment burden and fatigue, thereby improving adherence and preserving blinding quality. Regarding total pulse number, studies using <2000 pulses showed a significant reduction in VAS scores, whereas those using ≥2000 pulses did not. This pattern is broadly consistent with the findings reported by Attia et al. (19) and Zhang et al. (42). However, the apparent effect of pulse number may have been influenced by other correlated treatment characteristics, such as stimulation frequency and number of sessions, and should therefore be interpreted with caution. Notably, the pulse ranges in the two subgroups did not overlap, with 200–1500 pulses per treatment course in the <2000 subgroup and 2000–4000 pulses per treatment course in the ≥2000 subgroup, suggesting that these categories may reflect two distinct stimulation strategies used in clinical practice. Mechanistically, a moderate pulse load may regulate cortical excitability through long-term depression (LTD), whereas excessive stimulation could potentially induce neuronal overactivation and attenuate analgesic efficacy (43, 44).A similar pattern was observed for stimulation frequency. Compared with frequencies <10 Hz, frequencies ≥10 Hz were associated with significant reductions in VAS scores. This finding is in agreement with previous reports suggesting that 10–20 Hz may represent a potentially favorable range for analgesia (19, 42). High-frequency rTMS is generally considered to exert excitatory effects and may enhance synaptic plasticity through long-term potentiation (LTP), thereby restoring impaired cortical inhibitory control (45, 46). By contrast, low-frequency stimulation is more commonly associated with inhibitory effects and may reduce excessive cortical excitability through LTD-related mechanisms (43, 47). Importantly, rTMS may induce multiple forms of neuroplasticity simultaneously, allowing it to modulate abnormal pain-processing networks through more than one pathway (45). For stimulation intensity, the subgroup with a motor threshold of <90% RMT showed a significant reduction in VAS scores, whereas the ≥90% RMT subgroup did not. This result differs somewhat from the findings of Attia et al. (19), who suggested that stimulation intensities ranging from 80% to 120% RMT may be effective for analgesia. Such discrepancies may be related to differences in patient characteristics, study design, or methods used to determine RMT, all of which could influence treatment delivery and even the adequacy of blinding (48). Among all subgroup factors, stimulation at non-M1 targets was associated with a greater improvement in VAS scores than stimulation at the M1 target in patients with neuropathic pain, and the between-subgroup difference reached statistical significance. However, this finding is not entirely consistent with the majority of previous studies that have regarded M1 as the classical analgesic target for neuropathic pain; therefore, it should be interpreted with caution and should not be taken as evidence that non-M1 targets are generally superior to M1 (20). Several explanations may account for this discrepancy. First, the non-M1 subgroup in the present study did not represent a single brain region, but rather comprised multiple functionally heterogeneous targets, such as the dorsolateral prefrontal cortex (DLPFC) and other non-M1 regions. Different brain regions may participate in pain modulation through distinct mechanisms. For example, the DLPFC may primarily influence the affective and cognitive dimensions of pain, whereas other cortical regions may act through sensory integration or descending pain modulatory pathways (22, 28, 29). Second, the effect observed in the non-M1 subgroup may not reflect the true efficacy of any single target, but rather the combined influence of several different stimulation sites. In addition, the etiology and severity of neuropathic pain varied across the included studies. Peripheral and central neuropathic pain may differ in pathophysiological mechanisms, pain characteristics, and responsiveness to neuromodulatory interventions (49, 50). Third, the observed difference between the M1 and non-M1 subgroups may partly reflect imbalances in patient composition rather than an independent effect of stimulation site itself. Moreover, different targets may have been accompanied by differences in stimulation frequency, stimulation dose, total pulse number, and intervention period, all of which may independently affect analgesic outcomes (51–53). Fourth, because the number of included studies was limited, the pooled estimate for the non-M1 subgroup may have been influenced by a small number of studies or by specific neuropathic pain subtypes, thereby reducing the statistical stability of the result. Therefore, this finding is better regarded as exploratory, suggesting that brain regions other than M1 may have potential therapeutic value in selected neuropathic pain populations, but it remains insufficient to challenge the role of M1 as the classical stimulation target. Future randomized controlled trials with larger sample sizes, clearer target definitions, and more consistent parameter settings are needed to further clarify the true differences in efficacy between stimulation sites across different neuropathic pain subtypes.

However, formal tests for subgroup differences across intervention period, total pulse number, stimulation frequency, and motor threshold were not statistically significant. Accordingly, these findings should not be taken to indicate that any specific stimulation parameter is inherently superior to its comparator. As emphasized in the Cochrane Handbook, a true subgroup effect cannot be inferred solely from the observation that one subgroup reaches statistical significance whereas another does not; rather, such an inference should rely primarily on direct between-subgroup comparisons (54). Therefore, the present findings suggest only that analgesic effects were observed in certain subgroup strata, while the available evidence remains insufficient to establish true effect modification by intervention duration, pulse number, stimulation frequency, or stimulation intensity. These subgroup findings should be regarded as exploratory, as they may have been influenced by variations in sample size, statistical power, study distribution, and residual clinical heterogeneity. Moreover, the use of multiple subgroup comparisons increases the likelihood of chance findings. Future large-scale, well-designed randomized controlled trials employing more standardized stimulation protocols are needed to determine whether specific rTMS parameters are associated with differential effects on pain outcomes.

Although the overall results of this study suggest that rTMS may improve VAS scores in patients with NP, this conclusion should be interpreted with caution. The publication bias analysis indicated a possible risk of bias for this outcome, and the possibility that studies reporting negative results were less likely to be published cannot be ruled out. Such bias may have affected the precision of the pooled estimate and reduced the certainty of the available evidence. Accordingly, the observed improvement in VAS should be regarded as preliminary evidence supporting the analgesic potential of rTMS rather than definitive proof, and confirmation from larger, high-quality randomized controlled trials remains necessary. Several limitations should also be taken into account. First, the number of included studies was relatively small, and the overall sample size was limited, which may have reduced the stability of the pooled estimates; consequently, the subgroup findings should be considered exploratory. Second, substantial heterogeneity was observed for the VAS outcome. Although meta-regression and subgroup analyses were conducted, the underlying sources of this heterogeneity could not be fully identified. Third, potential publication bias may have further weakened the robustness of the evidence for some outcomes. Fourth, none of the included studies provided long-term follow-up data, making it impossible to determine whether the analgesic effects of rTMS can be sustained over time in patients with NP. Fifth, the generally high risk of bias in the included studies may have compromised the reliability of the pooled findings. In addition, the molecular mechanisms through which rTMS exerts its effects on NP remain insufficiently understood, which may limit its broader clinical translation. Further research is therefore warranted to clarify the molecular basis of rTMS in the treatment of NP. In summary, the findings of the present study should be interpreted prudently. Further well-designed RCTs with larger sample sizes, more standardized stimulation protocols, and longer follow-up periods are needed to verify and extend the current evidence.

## 5 Conclusion

rTMS exhibits beneficial effects in patients with NP. Subgroup analyses reveal that changes in VAS scores are significantly influenced by total pulse number, stimulation frequency, and stimulation site, whereas intervention period and intensity exert less impact. However, given the limited number of available studies, further research employing rigorous evaluation criteria and high-quality randomized controlled trials is necessary to better clarify the therapeutic potential of rTMS in this population.

## Data Availability

The original contributions presented in the study are included in the article/supplementary material. Further inquiries can be directed to the corresponding author.
